# A Family of Laminin α2 Chain-Deficient Mouse Mutants: Advancing the Research on LAMA2-CMD

**DOI:** 10.3389/fnmol.2020.00059

**Published:** 2020-04-21

**Authors:** Kinga I. Gawlik, Madeleine Durbeej

**Affiliations:** Muscle Biology Unit, Department of Experimental Medical Science, Lund University, Lund, Sweden

**Keywords:** muscular dystrophy, laminin, transgene, knockout, basement membrane, animal model

## Abstract

The research on laminin α2 chain-deficient congenital muscular dystrophy (LAMA2-CMD) advanced rapidly in the last few decades, largely due to availability of good mouse models for the disease and a strong interest in preclinical studies from scientists all over the world. These mouse models continue to provide a solid platform for understanding the LAMA2-CMD pathology. In addition, they enable researchers to test laborious, necessary routines, but also the most creative scientific approaches in order to design therapy for this devastating disorder. In this review we present animals belonging to the laminin α2 chain-deficient “*dy/dy*” mouse family (*dy/dy*, *dy*^2J^/*dy*^2J^, *dy*^3K^/*dy*^3K^, *dy*^W^/*dy*^W^, et al.) and a summary of the scientific progress they facilitated. We also raise a few questions that need to be addressed in order to maximize the usefulness of laminin α2 murine mutants and to further advance the LAMA2-CMD studies. We believe that research opportunities offered by the mouse models for LAMA2-CMD will continuously support our efforts to find a treatment for the disease.

## Introduction

The use of animals for scientific purposes goes back to ancient Greece where philosophers and scientists such as Alcmaeon of Croton, Aristotle, and Erasistratus dissected animals for anatomical studies. Today, the use of animals is a general practice for studying human biology and disease as the genetic similarity between humans and mice is very high. Also, the rapid development of methods for creating genetically modified animals increases their scientific value and stimulates general interest in preclinical research in society. One of the mouse models for laminin α2 chain-deficiency, the spontaneous *dy/dy* mutant, was described in 1955. It was the first reported mouse strain representing the pathological criteria that were characteristic for muscular dystrophy. The hereditary pattern of the defects suggested an autosomal recessive disorder and the causative mutation was called *dystrophia muscularis*, designated by the symbol *dy* (Michelson et al., 1955). Understandably, at that time it was not linked to any specific inherited muscle disease but in 1994 two independent research groups suggested that deficiency of laminin α2 chain might be the primary defect in *dy/dy* mice (Sunada et al., 1994; Xu et al., 1994a). Yet, until this date the mutation in the *Lama2* gene in the *dy/dy* mouse has not been identified.

## Overview of LAMA2-CMD Mouse Models

More than 300 mutations in the *LAMA2* gene have been identified so far in patients (Oliveira et al., 2018), which result in a large clinical heterogeneity of the disease. Realistically, the whole range of human mutations cannot be mimicked in mice, but currently available mouse models mirror well the broad spectrum of the human LAMA2-CMD condition (Table 1). This creates an opportunity for more efficient preclinical testing and conclusive preclinical studies.

**TABLE 1:** Summary of available LAMA2-CMD mouse models.

**Mouse**	**Mutation**	**Laminin α2 expression**	**Phenotype^#^**	**Time of death**	**References**
*dy/dy*	Spontaneous, unknown	Reduced expression of normal sized laminin α2	Moderate muscular dystrophy Peripheral neuropathy	Before 6 months of age	Sunada et al., 1994; Xu et al., 1994a
*dy*^2J^/*dy*^2^	Spontaneous splice site mutation in LN domain	Slightly reduced expression of truncated laminin α2 missing LN domain	Mild muscular dystrophy Peripheral neuropathy	After 6 months of age	Xu et al., 1994b; Sunada et al., 1995
*dy*^6J^/*dy*^6J^	Spontaneous, unknown	Unknown	Moderate (?) muscular dystrophy Peripheral neuropathy	Before 6 months of age	https://www.jax.org/strain/003589
*dy*^7J^/*dy*^7J^	ENU-induced missense mutation in LN domain	Normal levels of normal sized laminin α2	Mild muscular dystrophy Peripheral neuropathy	After 6 months of age	Patton et al., 2008
*dy*^W^/*dy*^W^	Knock-out	Severely reduced expression of truncated laminin α2 missing LN domain	Severe muscular dystrophy Peripheral neuropathy	5–12 weeks of age	Kuang et al., 1998b; Willmann et al., 2017
*dy*^3K^/*dy*^3K^	Knock-out	Complete deficiency	Very severe muscular dystrophy Peripheral neuropathy	3 weeks of age	Miyagoe et al., 1997
*dy*^8J^/*dy*^8J^ (extinct)	Spontaneous, unknown	Unknown	Severe (?) muscular dystrophy Peripheral neuropathy	3–4 weeks of age	https://www.jax.org/strain/009692
*dy*^Pas^/*dy*^Pas^ (extinct)	Spontaneous, retrotransposal insertion between exon 34 and 35	Complete deficiency	Severe muscular dystrophy Peripheral neuropathy	Before 13 weeks of age	Besse et al., 2003

*The symbol “#” denotes description of muscular dystrophy severity (mild, moderate, severe, and very severe). The severity is based on age of onset, histopathological severity and time of death. The symbol “?” denotes characterization of mice remains to be published. Description of severity is based on information on https://www.jax.org/.*

**FIGURE 1:**
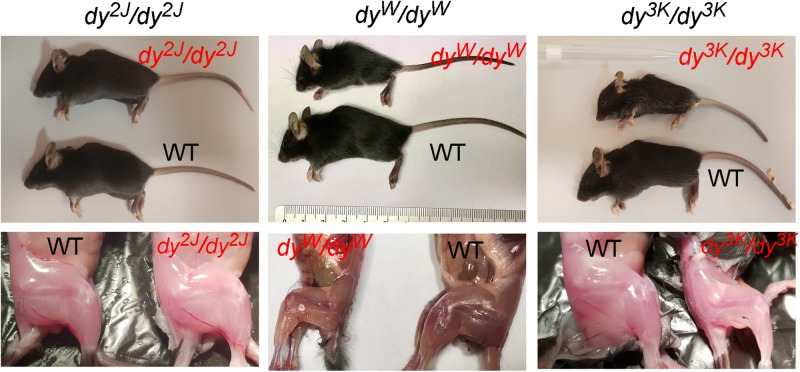
General phenotype of dystrophic LAMA2-CMD mice (*dy*^2J^/*dy*^2J^, *dy*^W^/*dy*^W^ and *dy*^3K^/*dy*^3K^) and their normal littermates (WT) at 3 weeks of age. Muscle wasting and weight loss are evident in *dy*^W^/*dy*^W^ and *dy*^3K^/*dy*^3K^ mice.

The three most commonly used animal models for LAMA2-CMD are *dy*^2J^/*dy*^2J^ (Xu et al., 1994b; Sunada et al., 1995), *dy*^3K^/*dy*^3K^ (Miyagoe et al., 1997), and *dy*^W^/*dy*^W^ mice (Kuang et al., 1998b; Table 1 and Figure 1). Skeletal muscle and peripheral nerve are the tissues with the most evident pathology in LAMA2-CMD mutants (Yurchenco et al., 2017). The dystrophic symptoms and general muscle pathology at the advanced stages of the disease have been fairly well characterized in all three models (Xu et al., 1994b; Miyagoe et al., 1997; Kuang et al., 1998b; Moll et al., 2001; Guo et al., 2003; Girgenrath et al., 2004, 2009; Gawlik et al., 2010, 2018; Carmignac et al., 2011a; Kumar et al., 2011; Pasteuning-Vuhman et al., 2018). Yet, we still need to learn more about mechanisms driving the disease progression in mouse. Only recently the importance of the embryonic, pre-symptomatic stages and early pathogenesis in the different mouse models for LAMA2-CMD has been emphasized (Gawlik et al., 2014, 2019; Mehuron et al., 2014; Nunes et al., 2017; Moreira Soares Oliveira et al., 2018). Likewise, associated symptoms in non-muscle tissues (peripheral and central nervous system, cardiorespiratory system) have gained attention (Hager et al., 2005; Qiao et al., 2005, 2018; Yang et al., 2005; Gawlik et al., 2006a, 2018, Homma et al., 2011; Menezes et al., 2014; Willmann et al., 2017; Pasteuning-Vuhman et al., 2018; Rabie et al., 2019).

Below we present a concise overview of the pathology in the three mouse models.

### Phenotype of *dy*^2J^/*dy*^2J^ Mice

**FIGURE 2:**
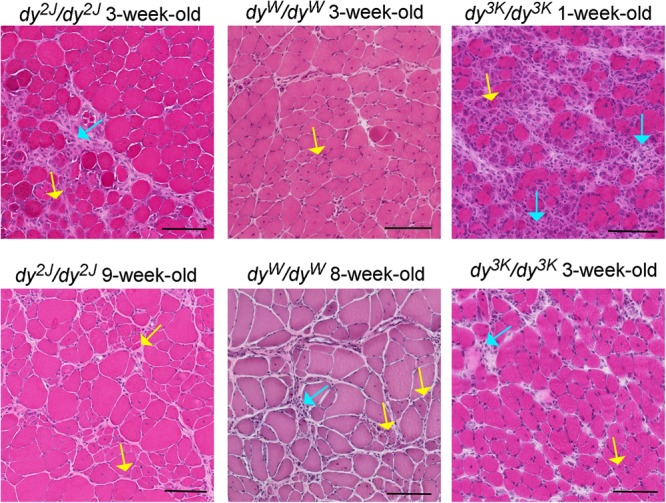
Different stages of muscle pathology in three LAMA2-CMD mouse models (*dy*^2J^/*dy*^2J^, *dy*^W^/*dy*^W^, and *dy*^3K^/*dy*^3K^). Limb muscles (rectus femoris for *dy*^2J^/*dy*^2J^ and *dy*^3K^/*dy*^3K^, triceps for *dy*^W^/*dy*^W^) were stained with hematoxylin and eosin. Yellow arrows point to regenerating fibers with centrally located nuclei; turquoise arrows depict areas with inflammatory cells. Bars, 100 μm.

*dy*^2J^/*dy*^2J^ mice carry a splice site mutation in the LN domain, which results in production of a shorter laminin α2 chain lacking the N-terminal portion of the molecule (Xu et al., 1994b; Sunada et al., 1995). The truncation hinders polymerization of laminin matrices and formation of basement membranes (Colognato and Yurchenco, 1999; Yurchenco et al., 2004; Yurchenco and Patton, 2009), but the phenotype of *dy*^2J^/*dy*^2J^ mice is relatively mild (Guo et al., 2003) (phenotype overview: Figures 1, 2 and Tables 2, 3). Their survival extends over 6 months of age (Xu et al., 1994b). Although the majority of dystrophic features are similar between *dy*^2J^/*dy*^2J^ males and females (dystrophic features in different muscle types) (Pasteuning-Vuhman et al., 2018), one has to take into consideration that gender-related phenotype differences (weight gain, creatine kinase levels (CK), water intake, muscle strength), exist in this mouse model (Fontes-Oliveira et al., 2018; Moreira Soares Oliveira et al., 2018; Pasteuning-Vuhman et al., 2018), which thus far has not been demonstrated for other LAMA2-CMD mutants. While these differences do not impact the overall disease presentation (they are rather subtle and a more severe phenotype cannot be attributed to any gender), they could significantly influence the outcomes of therapeutic strategies (Fontes-Oliveira et al., 2018). Hence, analysis of the treatment effects in *dy*^2J^/*dy*^2J^ mice should take into account both genders separately.

**TABLE 2:** An overview of time-points describing the disease progress in LAMA-CMD mouse models (*dy*^2J^/*dy*^2J^, *dy*^W^/*dy*^W^, *dy*^3K^/*dy*^3K^) based on muscle pathology.

	**Disease progress (age)**			
	
**Mouse model**	**No symptoms**	**Onset**	**Early disease**	**Established disease**
*dy*^2J^/*dy*^2J^	Time-point not determined	Time-point not determined	Week 3–4	Week 6 onward
*dy*^W^/*dy*^W^	E10.5–E17	E17.5–E18.5, smaller muscles	Week 1–2	Week 3–15
*dy*^3K^/*dy*^3K^	E18.5 (earlier time-points were not analyzed)	Postnatal day 1, apoptosis	Week 1	Week 2–3, despite improvements of muscle morphology (plateau)

*Onset: the first signs of muscle pathology. Early disease: muscle damage, inflammation and regeneration. Established disease: fibrosis, atrophy in addition to hallmarks mentioned earlier. Please note that only a few muscles were included in most of the studies and often a limited number of phenotyping methods was applied.*

**TABLE 3:** A summary of the overall health and muscle phenotype of LAMA2-CMD mouse models (*dy*^2J^/*dy*^2J^, *dy*^W^/*dy*^W^, *dy*^3K^/*dy*^3K^) at the different stages of development.

	**Developmental stage**				
	
**Mouse model**	**Embryonic**	**Neonatal, early postnatal (1–7 day old)**	**Postnatal (1–3 week-old)**	**Early adulthood (3–6 week-old)**	**Adulthood (older than 6 weeks)**
*dy*^2J^/*dy*^2J^	Not determined.	Not determined.	Week 1–2 not determined. Pronounced inflammation and increased CK at week 3.	Central nucleation, inflammation, muscle fiber size variation, weight loss, CK variable, impaired muscle function. Phenotype clearly visible outwardly.	Fibrosis, central nucleation, fiber size variation. Impaired neuromuscular function.
*dy*^W^/*dy*^W^	Normal back muscle morphology, smaller muscle size from E18.5, more detailed studies needed.	Smaller back muscles, limb muscles normal size. Morphology abnormalities, apoptosis, inflammation.	Atrophy (smaller limb muscles, muscle fiber loss), apoptosis, central nucleation, inflammation, fibrosis onset. Phenotype clearly visible outwardly.	Apoptosis, inflammation, central nucleation, muscle fiber loss, fibrosis, increased CK, muscle function impairment.	Apoptosis, inflammation muscle fiber loss, atrophy, fibrosis, increased CK, muscle function impairment.
*dy*^3K^/*dy*^3K^	Normal limb muscle, more detailed studies needed.	Apoptosis, muscle degeneration, muscle fiber loss, inflammation, central nucleation, weight loss. Phenotype clearly visible outwardly.	Muscle repair, atrophy (smaller muscle fibers), apoptosis, inflammation, increased extracellular matrix deposition. Impaired motor function. Abnormalities of masticatory muscles. Increased CK, death at week 3.	Currently NA (death at age of 3 weeks). In previous studies when *dy*^3K^/*dy*^3K^ mice survived up to 5 weeks: Apoptosis, inflammation, central nucleation, muscle fiber loss, fibrosis, increased CK, muscle function impairment.	NA (death at age of 3 weeks).

*NA – not applicable.*

The pre-symptomatic stages of the disease in the *dy*^2J^/*dy*^2J^ mouse have not been delineated. The earliest time-point analyzed is 3 weeks of age and at this stage *dy*^2J^/*dy*^2J^ animals show normal muscle strength and collagen content in muscle is not changed (Moreira Soares Oliveira et al., 2018). However, muscle damage has already taken place as demonstrated by elevated CK levels, pronounced inflammatory response and occurrence of regenerating fibers (Figure 2; Kemaladewi et al., 2018; Moreira Soares Oliveira et al., 2018). Additionally, 3-week-old *dy*^2J^/*dy*^2J^ males weigh less than wild-type male littermates. One week later, other dystrophic hallmarks (central nucleation, decreased muscle strength) are established and fully developed pathology can be observed at 6–8 weeks of age, including increased production of several extracellular matrix components, muscle atrophy and decreased body weights in both genders (Nevo et al., 2010; McKee et al., 2017; Fontes-Oliveira et al., 2018; Moreira Soares Oliveira et al., 2018; Tables 2, 3). Reduced muscle strength and mobility between 4–10 weeks of age has been clearly documented in several studies (Dadush et al., 2010; McKee et al., 2017; Fontes-Oliveira et al., 2018; Gawlik et al., 2018; Moreira Soares Oliveira et al., 2018; Pasteuning-Vuhman et al., 2018). The follow-up of the *dy*^2J^/*dy*^2J^ mouse condition up to 34 weeks of age did not reveal significant muscle pathology progression compared to 6-week-old mice (Pasteuning-Vuhman et al., 2018), except for the additional body weight loss between week 6–10 (Fontes-Oliveira et al., 2018). In contrary, the CK levels were normalized with age (Holmberg et al., 2014; Pasteuning-Vuhman et al., 2018) and challenging the mice with functional tests throughout the course of the disease (between week 6 and 34) did not worsen the dystrophic symptoms (Pasteuning-Vuhman et al., 2018). It is also important to mention that different muscles display variation in the severity of the phenotype (gastrocnemius and tibialis anterior are more affected than triceps; diaphragm shows mild pathology) and distinct patterns of pathology development (for example different timing and degree of inflammation) (Gawlik et al., 2018; Kemaladewi et al., 2018; Pasteuning-Vuhman et al., 2018).

Due to its prolonged survival time compared to other mouse models, the *dy*^2J^/*dy*^2J^ mouse is widely used for studies of peripheral neuropathy associated with laminin α2 chain-deficiency (Yang et al., 2005; Gawlik et al., 2018; Rabie et al., 2019). The first signs of peripheral neuropathy (hind limb clasping when lifted by tail) are visible 4 weeks after birth and aggravate with age. Temporary hind limb paralysis (symptoms ceasing and relapsing, often in one limb only) occurs already around week 6 and permanent hind limb paralysis by 3 months of age (Yang et al., 2005) (it is noteworthy that forelimbs, at least outwardly, are not affected). These symptoms are caused by impaired axonal sorting and dysmyelination (Yang et al., 2005; Gawlik et al., 2018), particularly in motor nerves (ventral roots), but also in dorsal roots (Yang et al., 2005; Rabie et al., 2019). Accordingly, motor nerve conduction velocity in the sciatic nerve is reduced (Domi et al., 2015; Rabie et al., 2019). It is important to mention that peripheral nerve defects cause neurogenic atrophy of muscle fibers (Gawlik et al., 2004; McKee et al., 2012) and contribute to dystrophic phenotype of skeletal muscle. Interestingly, although peripheral neuropathy is pronounced in all LAMA2-CMD mouse models (Guo et al., 2003), it is rarely manifested in patients (Yurchenco et al., 2017).

Respiratory function in *dy*^2J^/*dy*^2J^ mice has been assessed with the whole-body plethysmography. The respiratory rate and amplitude were significantly impaired in *dy*^2J^/*dy*^2J^ mice and the respiration rate further declined with age (Yu et al., 2013; Pasteuning-Vuhman et al., 2018). Cardiac manifestations are associated with some cases of LAMA2-CMD (mostly attributed to complete absence of laminin α2 chain) (Nguyen et al., 2019). Cardiomyopathy has not been observed in *dy*^2J^/*dy*^2J^ mice as assessed by histological means (8-week-old animals) (Gawlik et al., 2018) and echocardiography (12–15 and 30–33 week-old animals) (Yu et al., 2013). Yet, the heart rates are increased in *dy*^2J^/*dy*^2J^ mutants (Yu et al., 2013) and hearts from 10-week-old dystrophic mice tend to weigh less (Fontes-Oliveira et al., 2018) than wild-type hearts. In the light of growing evidence that cardiac defects in patients (also with partial deficiency of laminin) could be underreported in literature (Nguyen et al., 2019), it is possible that histological features of cardiomyopathy may be manifested in hearts from older *dy*^2J^/*dy*^2J^ animals, despite no clear changes in electrophysiological parameters.

### Phenotype of *dy*^W^/*dy*^W^ Mice

The *dy*^W^/*dy*^W^ mouse was generated by homologous recombination in embryonic stem cells and intended to represent the *Lama2-*null mutant (Kuang et al., 1998a, b). It was revealed later that the mouse is not completely devoid of laminin α2 chain, but shows strongly reduced expression of the truncated molecule (lacking the LN domain) (Guo et al., 2003). The phenotype is severe and *dy*^W^/*dy*^W^ mice die typically 5–12 weeks after birth (Kuang et al., 1998b) (phenotype overview: Figures 1, 2 and Tables 2, 3), but some do not survive post-weaning^1^.

Early timepoints of the disease pathology have been fairly well characterized in this mouse model (Mehuron et al., 2014; Nunes et al., 2017), including embryonic stages (E10.5–E18.5) (Nunes et al., 2017). In fact, myogenesis defects *in utero* have been pinpointed in *dy*^W^/*dy*^W^ mice (Nunes et al., 2017). While no obvious defects of muscle development have been demonstrated in LAMA2-CMD before (Kuang et al., 1998a; Gawlik and Durbeej, 2011), myofibers are smaller at birth and muscle degeneration/regeneration occurs shortly after birth in patients (Hayashi et al., 2001; Voit and Tome, 2004). Additionally, reduction of fetal movements has been reported (Jones et al., 2001). Hence, it is reasonable to consider muscle developmental abnormalities in LAMA2-CMD (even if they are subtle).

Although myotomal (E10.5), primary (E11.5–E13.5) and the first stages of secondary myogenesis (E14.5–E16.5) proceed normally in *dy*^W^/*dy*^W^ mice, the final stages of fetal myogenesis (E17.5–E18.5) are marked with a few abnormalities (studied in epaxial muscles). The developmental defects include decreased number of Pax7 and myogenin positive cells (secondary myogenesis, E17.5 onward), smaller muscles (E18.5 onward), which results in muscle growth impairment that is not recovered during postnatal development (Nunes et al., 2017; Tables 2, 3).

A few discrepancies concerning early postnatal development emerge from two separate studies: Nunes et al. (2017) reported no obvious morphology change in 2-day-old *dy*^W^/*dy*^W^ muscle, but alteration of muscle size (epaxial muscles), whereas Mehuron et al. (2014) observed morphology defects in 1-day-old muscle with clearly reduced number of myofibers, but no change in muscle size (tibialis anterior). This suggests that the characteristics of pathological events could differ between muscles and one has to be careful to draw generalized conclusions when studying one muscle. Accordingly, it has been shown in older mice that tibialis anterior is more affected than triceps (Reinhard et al., 2017), soleus and extensor digitorum (Vohra et al., 2015).

The natural history of the disease studied in *dy*^W^/*dy*^W^ limb muscles between 1 and 4-weeks of age reveals appearance of a broad range of dystrophic hallmarks (Figure 2 and Tables 2, 3. One-week old *dy*^W^/*dy*^W^ muscle is characterized with muscle fiber loss, extensive apoptosis, initiation of the inflammatory response together with the myogenic program as well as increased production of fibronectin, osteopontin and matrix remodeling proteins (Wardrop and Dominov, 2011; Mehuron et al., 2014). A decreased number of myofibers does not correlate with loss of body weight, muscle weight, and reduction of muscle size (Mehuron et al., 2014), although slight body weight differences between wild-type mice and *dy*^W^/*dy*^W^ mutants have been reported for 1-week-old animals in another study (Wardrop and Dominov, 2011). Upregulation of fibrillar collagens accompanies the pathology progression at week 2. Decrease in limb muscle weight coincides with the delay of the whole-body weight gain at week 3 (Mehuron et al., 2014). CK is significantly elevated in serum from 1-month-old *dy*^W^/*dy*^W^ mice (Kuang et al., 1998b) and fibrotic lesions are a signature of advanced pathology in *dy*^W^/*dy*^W^ muscle (Meinen et al., 2012; Accorsi et al., 2015; Accorsi et al., 2016). Interestingly, apoptosis is less pronounced in 3–4 week-old mice compared to younger mutants (Mehuron et al., 2014). Basement membranes have patchy appearance in *dy*^W^/*dy*^W^ muscle (Moll et al., 2001). The dystrophic changes described above correlate with impaired muscle function (Moll et al., 2001; Doe et al., 2011; Willmann et al., 2017).

Peripheral neuropathy is a prominent feature of the disease also in the *dy*^W^/*dy*^W^ mouse model (Kuang et al., 1998b), just like in all mouse models for LAMA2-CMD (Yurchenco et al., 2017). Several studies have explored the phenotype of peripheral nervous system in *dy*^W^/*dy*^W^ mutant, showing myelination defects in sciatic nerve and ventral roots, smaller cross sectional area of sciatic nerve, as well as motor dysfunction and sensorimotor gating deficits at age of 4–6 weeks (Homma et al., 2011; Qiao et al., 2018).

The respiratory function in *dy*^W^/*dy*^W^ mice has also been evaluated by whole-body plethysmography. However, the breath rate did not appear to change over time and may not be predictive of pathology in *dy*^W^/*dy*^W^ animals (Willmann et al., 2017). Analysis of cardiac muscle in this mouse model is required.

### Phenotype of *dy*^3K^/*dy*^3K^ Mice

The *dy*^3K^/*dy*^3K^ mouse is the only available LAMA2-CMD mouse model void of laminin α2 subunit (Miyagoe et al., 1997; Guo et al., 2003). The complete loss of laminin α2 chain conceivably contributes to the most severe phenotype and much earlier death of the *dy*^3K^/*dy*^3K^ mouse compared to other models (currently 3 weeks of age, they used to survive up to 5 weeks, but their phenotype has gradually worsened over the years in our colony at Lund University) (phenotype overview: Figures 1, 2, Tables 2, 3, and Supplementary Video 1). The cause of death is most probably linked to malnutrition and respiratory difficulties (Gawlik et al., 2019).

No obvious development abnormalities have been detected in embryonic *dy*^3K^/*dy*^3K^ muscle (E18.5, calf muscles, thigh muscles) (Gawlik et al., 2019). Yet, more detailed studies are warranted in a wide range of muscles to confirm the current observations and comprehend myogenic events in *dy*^3K^/*dy*^3K^ embryos. One-day-old *dy*^3K^/*dy*^3K^ muscles display normal morphology (fiber size, number of fibers). Inflammation and abnormal expression of the extracellular matrix components have not been detected. Yet, occasional occurrence of apoptotic fibers marks the pathogenesis onset in 1-day-old *dy*^3K^/*dy*^3K^ muscle (Gawlik et al., 2019). This suggests that *dy*^3K^/*dy*^3K^ muscles are prone to damage already at the neonatal stage, when the mouse movements are limited to sporadic limb waddling. Muscle damage progresses quickly and already 4-day-old muscles show inflammatory response to injury. At day 7 the body weight is significantly reduced coinciding with pronounced loss of muscle fibers and inflammation (Figure 2; Miyagoe et al., 1997; Gawlik et al., 2019). The extracellular matrix components are massively upregulated, which is associated with muscle repair. Importantly, the severe phenotype observed at day 7 is partially ameliorated with age: at day 14 and 21 *dy*^3K^/*dy*^3K^ muscles show recovery of muscle fiber number, reduced inflammation (Figure 2) and downregulation of fibronectin and collagen III (Gawlik et al., 2019). Yet, apoptotic fibers and regenerating fibers are present at these stages (Miyagoe et al., 1997; Gawlik et al., 2019). Despite normalization of the number of myofibers, there is a decrease in fiber size and muscle mass (Carmignac et al., 2011a; Gawlik et al., 2019). Likewise, although the abundance of interstitial extracellular matrix is diminished in older mutants, the levels of collagen III and fibronectin remain elevated compared to a healthy age-matched muscle (Table 3). CK levels are also increased in serum at that stage compared to wild-type mice (Gawlik et al., 2010; Holmberg et al., 2014). Basement membranes surrounding muscle fibers are discontinuous throughout the disease course (Miyagoe et al., 1997; Gawlik et al., 2004). Similarly to other LAMA2-CMD mouse models, *dy*^3K^/*dy*^3K^ limb muscles display variety of phenotype presentation: vastus lateralis, medialis, gastrocnemius are more affected than rectus femoris, triceps and tibialis anterior (Gawlik et al., 2019). However, one has to be aware that complete quantitative analyses of dystrophic features in a wide range of muscles over time are missing for all mouse models.

Interestingly, the muscle function tests performed on *dy*^3K^/*dy*^3K^ mice at different ages do not necessarily correlate with muscle phenotype. For example, 7-day-old *dy*^3K^/*dy*^3K^ animals do not display significant difficulties when performing functional tests. On the contrary, 14-day-old mice show significant impairments of motor function (Gawlik et al., 2019).

Non-limb muscles have been studied in the *dy*^3K^/*dy*^3K^ mouse (Nystrom et al., 2006; Hager and Durbeej, 2009; Gawlik et al., 2014, 2019). The principal extraocular muscle and intrinsic laryngeal muscles appear spared (Nystrom et al., 2006; Hager and Durbeej, 2009), whereas accessory extraocular muscles show signs of myopathy (Gawlik et al., 2014). Esophagus shows mild muscular dystrophy (day 21); diaphragm, intercostal muscles, tongue, and temporalis demonstrate moderate dystrophic changes (studied at day 14 and 21). The most severe phenotype has been observed in masseter muscle (day 7, 14, and 21) (Gawlik et al., 2014, 2019).

Heart muscle has not been shown to be affected in *dy*^3K^/*dy*^3K^ mouse, based on histology analysis (Gawlik et al., 2004; Gawlik and Durbeej, 2015). It might be that *dy*^3K^/*dy*^3K^ animals die too early to develop cardiac manifestations. Alternatively, the presence of laminins containing laminin α4 chain that are expressed in *dy*^3K^/*dy*^3K^ heart (Durbeej lab, unpublished data) might be sufficient to prevent cardiomyopathy in laminin α2-chain deficient mice. However, it is not excluded that cardiac function could be impaired and this needs to be verified by electrophysiology studies.

It is interesting that death of *dy*^3K^/*dy*^3K^ mouse coincides with the improvement of limb muscle phenotype, which points toward severe defects in other tissues, importance of masticatory muscles for proper nutrition, and perhaps importance of respiration maintenance. Respiratory function is yet to be analyzed in *dy*^3K^/*dy*^3K^ mice.

Demyelination of axons in sciatic nerve and in particular in ventral and dorsal roots, as well reduced size of axons (sciatic nerve) have been reported in *dy*^3K^/*dy*^3K^ mice (Nakagawa et al., 2001; Gawlik et al., 2006a). These defects result in reduced motor nerve conduction velocity of sciatic nerve (Nakagawa et al., 2001). Despite that, hind limb paralysis is not clearly manifested, probably due to early death of *dy*^3K^/*dy*^3K^ animals. Additionally, epilepsy episodes have been observed (Durbeej lab, unpublished observations).

### Other Mouse Models

The moderately affected *dy/dy* mouse expresses reduced amounts of full-length laminin α2 subunit (Sunada et al., 1994; Xu et al., 1994a). Patients showing a similar expression defect exist (Nissinen et al., 1996; Voit and Tome, 2004). Therefore, *dy/dy* animals constitute an important addition to *dy*^2J^/*dy*^2J^, *dy*^3K^/*dy*^3K^, and *dy*^W^/*dy*^W^ murine models, especially because cardiomyopathy in *dy/dy* mouse has been clearly demonstrated (fibrotic lesions at week 8, hypertrophic cardiomyopathy as evidenced by echocardiography at week 18) (Rash et al., 1998; Gawlik et al., 2010). *Dy/dy* mice live between 12 and 24 weeks (or longer), weigh much less already at the age of 3 weeks and remain very weak throughout the disease course (Connolly et al., 2001). They also display peripheral neuropathy (Matsumura et al., 1997). Initially this mouse model greatly facilitated experimental studies of the molecular pathology of the disease (Sunada et al., 1994; Xu et al., 1994a; Ringelmann et al., 1999). Unfortunately, the unidentified mutation makes the model more difficult to study (genotyping is not possible, which negatively impacts breeding strategies). Hence, the use of *dy/dy* mice in research has diminished.

A point mutation (Arg to Cys) disrupting a conserved paired cysteine motif in *dy*^7J^/*dy*^7J^ mice results in mild pathology and normal laminin α2 levels (Patton et al., 2008). In contrast to the other LAMA2-CMD mouse models, basement membrane is thickened in *dy*^7J^/*dy*^7J^ muscle and normal in peripheral nerve. Yet, muscle degeneration and severe amyelination of nerve roots affect the *dy*^7J^/*dy*^7J^ mouse (Patton et al., 2008). This mouse model provides opportunities to investigate underlying mechanisms and treatments for mild forms of LAMA2-CMD and to study additional factors that could play major roles in modulating laminin-related interactions in basement membrane (Patton et al., 2008). In particular, *dy*^7J^/*dy*^7J^ mutant overthrows the dogma that the presence of basement membrane *per se* is necessary for prevention of the dystrophic phenotype.

Yet another mouse model, *dy*^6J^/*dy*^6J^, is available at Jackson Laboratories. The mutation remains to be identified but mice show progressive weakness and hind limb paralysis is visible at about 3 weeks of age^2^. Lastly, two nowadays extinct mouse models have also been described; *dy*^8J^/*dy*^8J^ and *dy*^Pas^/*dy*^Pas^. The former was identified at Jackson Laboratories^3^ and the latter spontaneously arose at the Pasteur Institute in Paris (Besse et al., 2003).

## Molecular Aspects of Pathogenesis Studied in LAMA2-CMD Models

It is difficult to study the pathogenesis of laminin α2 chain-deficiency *in vivo* in patients (ethical considerations, limited access to human material, large heterogeneity of patients). Mouse models for LAMA2-CMD help to overcome these obstacles and are excellent tools to explore molecular mechanisms of the disease. Over the years a tremendous amount of studies describing different aspects of the LAMA2-CMD pathology in mice has been collected. These include: (1) description of cellular and molecular events in laminin α2 chain-deficient muscle, (2) transgenic and gene therapy strategies to prevent the dystrophic phenotype (Table 4), (3) knockout strategies to prevent the dystrophic phenotype and/or understand the mechanisms of the disease (Table 5), and (4) pharmacological approaches to develop clinical treatment options (Table 6).

**TABLE 4:** Summary of LAMA2-CMD mice overexpressing transgenes or AAV/lentivirus.

**Approach**	**Transgene/AAV**	**Mouse model**	**Muscle condition and overall health**	**References**
Extracellular matrix and	Laminin α2	*dy*^W^/*dy*^W^	Improved	Kuang et al., 1998b
receptor modulation		*dy*^2J^/*dy*^2J^	Improved	
	Laminin α1	*dy*^3K^/*dy*^3K^	Improved	Gawlik et al., 2004, 2006a, 2018;
		*dy*^2J^/*dy*^2J^	Improved	Gawlik and Durbeej, 2010
	Truncated laminin α1	*dy*^3K^/*dy*^3K^	Improved	Gawlik et al., 2010
	AAV carrying dCas9, VP64 transactivators and single-guide RNAs targeting *Lama1* promoter.	*dy*^2J^/*dy*^2J^	Improved	Kemaladewi et al., 2019
	AAV carrying Cas9, and single-guide RNAs correcting the splicing defect	*dy*^2J^/*dy*^2J^	Improved	Kemaladewi et al., 2017
	Mini-agrin	*dy*^W^/*dy*^W^	Improved	Moll et al., 2001;
		*dy*^3K^/*dy*^3K^	Improved	Bentzinger et al., 2005
	Mini-agrin AAV1 and 9	*dy*^W^/*dy*^W^	Improved	Qiao et al., 2005, 2018
	Mini-agrin + Bcl-2	*dy*^W^/*dy*^W^	Improved	Meinen et al., 2011
	Mini-agrin + αLNNd	*dy*^W^/*dy*^W^	Improved	Reinhard et al., 2017
	Agrin	*dy*^W^/*dy*^W^	Improved	Meinen et al., 2007
	Agrin-perlecan	*dy*^W^/*dy*^W^	Improved	Meinen et al., 2007
	αLNNd	*dy*^2J^/*dy*^2J^	Improved	McKee et al., 2017
	Integrin α7	*dy*^W^/*dy*^W^	Improved	Doe et al., 2011
	Galgt2/galgt2 AAV	*dy*^W^/*dy*^W^	Improved	Xu et al., 2007
Apoptosis inhibition	Bcl-2	*dy*^W^/*dy*^W^	Improved	Girgenrath et al., 2004
Promoting regeneration	IGF-1	*dy*^W^/*dy*^W^	Improved	Kumar et al., 2011
	IGF-1 + Bax null	*dy*^W^/*dy*^W^	Improved	Yamauchi et al., 2013
	ADAM12	*dy*^W^/*dy*^W^	No difference	Guo et al., 2005
Polyamine modulation	Smox or Amd1 lentivirus	*dy*^2J^/*dy*^2J^	Improved	Kemaladewi et al., 2018
				

**TABLE 5:** Summary of LAMA2-CMD mice lacking other genes.

**Approach**	**Deletion**	**Mouse model**	**Muscle condition and overall health**	**References**
Extracellular matrix and receptor modulation	Integrin α7	*dy*^3K^/*dy*^3K^	No difference	Gawlik and Durbeej, 2015
	Dystrophin	*dy*^3K^/*dy*^3K^	Worsened	Gawlik et al., 2014
	β-sarcoglycan	*dy*^3K^/*dy*^3K^	Worsened	Gawlik et al., 2014
	Laminin α4	*dy*^3K^/*dy*^3K^	Overall phenotype worsened but skeletal muscle condition remains to be reported	Miner et al., 2004
		*dy*^2J^/*dy*^2J^		
Apoptosis inhibition	Bax	*dy*^W^/*dy*^W^	Improved	Girgenrath et al., 2004
Modulation mitochondrial permeability transition	Cyclophilin D	*dy*^W^/*dy*^W^	Improved	Millay et al., 2008
Inflammation and fibrosis modulation	Complement C3	*dy/dy*	Improved	Connolly et al., 2002
	Galectin-3	*dy*^3K^/*dy*^3K^	No difference	Gawlik et al., 2017
	Osteopontin	*dy*^3K^/*dy*^3K^	Slightly worsened	Gawlik et al., 2017
	miR-21	*dy*^3K^/*dy*^3K^	No difference	Moreira Soares Oliveira et al., 2017
		*dy*^2J^/*dy*^2J^	No difference	
Muscle growth	Myostatin	*dy*^W^/*dy*^W^	No difference	Li et al., 2005
				

**TABLE 6:** Summary of pharmacological approaches in LAMA2-CMD mice.

**Approach**	**Compound**	**Mouse model**	**Muscle condition and overall health**	**References**
Extracellular matrix modulation	Laminin-111 protein	*dy*^W^/*dy*^W^	Improved	Rooney et al., 2012
Apoptosis inhibition	Doxycycline	*dy*^W^/*dy*^W^	Improved	Girgenrath et al., 2009
	Omigapil	*dy*^W^/*dy*^W^	Improved	Erb et al., 2009; Yu et al., 2013
		*dy*^2J^/*dy*^2J^	Improved	
	Omigapil in combination with mini-agrin transgene	*dy*^W^/*dy*^W^	Improved	Meinen et al., 2011
Proteasome inhibition	MG-132	*dy*^3K^/*dy*^3K^	Improved	Carmignac et al., 2011a
	Bortezomib	*dy*^3K^/*dy*^3K^	Improved	Korner et al., 2014; Korner and Durbeej, 2016
		*dy*^2J^/*dy*^2J^	No difference	
Autophagy inhibition	3-methyladenine	*dy*^3K^/*dy*^3K^	Improved	Carmignac et al., 2011b
Inflammation and fibrosis modulation	Losartan	*dy*^2J^/*dy*^2J^	Improved	Elbaz et al., 2012; Vohra et al., 2015
		*dy*^W^/*dy*^W^	Improved	
	Losartan derivative	*dy*^W^/*dy*^W^	Improved	Meinen et al., 2012
	Losartan and IGF-1 transgene	*dy*^W^/*dy*^W^	Improved	Accorsi et al., 2016
	Losartan and growth hormone	*dy*^W^/*dy*^W^	Improved	Accorsi et al., 2016
	Halofuginone	*dy*^2J^/*dy*^2J^	Improved	Nevo et al., 2010
	Glatiramer acetate	*dy*^2J^/*dy*^2J^	Improved	Dadush et al., 2010
	Prednisolone	*dy/dy*	Improved	Connolly et al., 2002
Muscle growth	Clenbuterol	*dy/dy*	Improved	Hayes and Williams, 1998
	IPLEX	*dy*^W^/*dy*^W^;*Bax-/-*	Improved	Yamauchi et al., 2013
Calcium modulation	Caldecrin	*dy/dy*	Improved	Tomomura et al., 2011
Metabolism modulation	Metformin	*dy*^2J^/*dy*^2J^	Improved (in females)*	Fontes-Oliveira et al., 2018
Oxidative stress inhibition	N-acetyl-L-cystein, vitamin E	dy^2J^/dy^2J^	Improved	Harandi et al., 2020
Exon skipping	Phosphorodiamidate morpholino oligomer targeting exon 4 of *Lama2*	*dy*^3K^/*dy*^3K^	n.d.	Aoki et al., 2013

*Some minor beneficial effects were also noted in males. n.d., not determined.

### Cellular and Molecular Events in Murine Laminin α2 Chain-Deficient Muscle

Loss/reduction/truncation of laminin α2 subunit triggers secondary molecular changes in skeletal muscle. These changes lead to disruption of muscle homeostasis, but some of them may also constitute compensatory alterations. For example, a compensatory upregulation of laminin α4 and α5 subunits in LAMA2-CMD mouse models has been described (Ringelmann et al., 1999; Moll et al., 2001; Gawlik et al., 2004, 2018; McKee et al., 2017). Laminin α4 and laminin α5 chains do not bind equally well to α-dystroglycan compared to laminin α1 and α2 subunits (Talts et al., 1999, 2000; Ferletta et al., 2003) and laminin α4 chain is also devoid of a domain that is essential for laminin polymerization (Yurchenco and Patton, 2009). Therefore, this upregulation only partially prevents a more acute disease progression (Voit and Tome, 2004). Ablation of laminin α4 in *dy*^3K^/*dy*^3K^ and *dy*^2J^/*dy*^2J^ mice apparently worsened the phenotype (*dy*^3K^/*dy*^3K^ double mutant reported to die before 2 weeks of age) but the skeletal muscle phenotype remains to be characterized (Miner et al., 2004). Hence, the role of laminin α4 chain in LAMA2-CMD is not entirely clear. Yet, it is possible to enhance polymerization and muscle cell binding of compensatory laminin chains and this strategy has been used to boost the phenotype of laminin α2 chain-deficient mice (Moll et al., 2001; Meinen et al., 2007; McKee et al., 2017; Reinhard et al., 2017) (mini-agrin or agrin overexpression that re-establishes connection between laminin α4 chain and dystroglycan; overexpression of laminin/nidogen chimeric protein that provides polymerization domain and combinatorial approach using both mini-agrin and the chimeric protein, see also section “Laminin α2 Chain-Deficient Mice Overexpressing Transgenes or Lacking Other Genes”).

The laminin α2 chain receptor integrin α7 (forming a dimer with the β1 subunit) is lost from the sarcolemma of laminin α2 chain-deficient muscle (Vachon et al., 1997; Hodges et al., 1997; Cohn et al., 1999; Gawlik et al., 2004, 2006b; Doe et al., 2011). Dystroglycans, on the other hand (both α and β), are upregulated (Gawlik et al., 2006b). It is not excluded that other, yet unidentified, laminin receptors in muscle are also perturbed.

Impaired interactions between laminin α2 and its binding partners instigate alternations of signaling pathways. Mouse models for LAMA2-CMD have provided an excellent platform for studies of signaling events. Signaling cascades associated with apoptosis, inflammation, metabolism, regeneration, protein turnover, and fibrosis (GAPDH-Siah1-CBP/p300-p53, Akt, TGF-β, NFκB, p53, JAK/STAT, to mention a few) have been shown to be affected in laminin α2 chain-deficient murine muscle (Girgenrath et al., 2004, 2009; Erb et al., 2009; Carmignac et al., 2011a, b; Kumar et al., 2011; Meinen et al., 2012; Elbaz et al., 2015; de Oliveira et al., 2014; Mehuron et al., 2014; Accorsi et al., 2015; Fontes-Oliveira et al., 2017; Gawlik et al., 2017, 2019; Nunes et al., 2017; Pasteuning-Vuhman et al., 2018; Yoon et al., 2018). Additionally, microarray, RNA-sequencing and proteomic technologies were applied to study murine LAMA2-CMD dystrophic muscle and provided a global overview of the gene and protein expression changed upon laminin α2 chain-deficiency (van Lunteren et al., 2006; Hager et al., 2008; de Oliveira et al., 2014; Kemaladewi et al., 2017; Moreira Soares Oliveira et al., 2018; Yanay et al., 2019). Such findings are essential to capitalize on opportunities given by preclinical studies and advance toward treatment design.

Analysis of cellular events in dystrophic muscle comes hand in hand with the studies of molecular interactions. A lot of attention has been given to regeneration and cells that could repair damaged muscle (satellite cells, myoblasts, non-muscle cells with myogenic potential). The subset of proliferating pro-regenerative cells has been shown to be diminished in *dy*^W^/*dy*^W^ muscle (Girgenrath et al., 2005), resulting in myogenesis impairment and differentiation delay in laminin α2 chain-deficient muscle (Kuang et al., 1999; Kumar et al., 2011; Mehuron et al., 2014). Consequently, cell therapy approaches have been implemented in *dy/dy, dy^W^/dy^W^*, and *dy*^3K^/*dy*^3K^ mice to support muscle renewal (myoblast, bone marrow and CD90-positive cells transplantation, manipulated mesoangioblasts) (Vilquin et al., 1996, 1999; Hagiwara et al., 2006; Fukada et al., 2008; Domi et al., 2015). Further studies exploring the properties of adult stem cells in laminin α2 chain-deficient muscular dystrophy are warranted, especially in the light of the enormous impact of the basement membranes on the stem cell niche remodeling in muscle (Rayagiri et al., 2018).

Muscle regeneration, inflammation and fibrosis are tightly connected in muscular dystrophy and this venue has, to some extent, been explored in mouse models for LAMA2-CMD. There is a thin line between correct tissue repair and uncontrolled fibrosis. It is all about “getting the balance right” and the balancing factor is inflammation (Perdiguero et al., 2012; Kharraz et al., 2013; Serrano and Munoz-Canoves, 2017). Since fibrosis is a signature of LAMA2-CMD (Mrak, 1998; Voit and Tome, 2004; Elbaz et al., 2012; Mehuron et al., 2014; Accorsi et al., 2015), inflammation should take a central spot in aiming at disease prevention. Yet, not that much is known about the inflammatory response in LAMA2-CMD, but probably it is crucial for the initial wave of muscle repair (Gawlik et al., 2017, 2019). Monocytes, macrophages and neutrophils, the elements of innate immune response, constitute inflammatory infiltrates at the site of muscle damage in laminin α2 chain mutants (Connolly et al., 2002; Kumar et al., 2011; Wardrop and Dominov, 2011; Meinen et al., 2012; Gawlik et al., 2017). Also, cytokines de-regulation has been demonstrated in laminin α2 chain-deficient murine muscle (Wardrop and Dominov, 2011; Gawlik et al., 2017). Much less is known about adaptive immunity and role of lymphocytes in laminin α2 chain-deficiency, although a few T lymphocytes have been identified in laminin α2 chain-deficient muscle (Rooney et al., 2012). Even if fibrosis has a destructive impact on condition of dystrophic muscle, myofibroblasts and fibroblasts have not been studied in LAMA2-CMD mouse models.

In summary, more effort should be dedicated to decipher interactions between cells involved in muscle regeneration, inflammation and fibrosis in LAMA2-CMD mouse models. Such studies could provide answers to mechanisms of human pathology and identify molecular targets for therapy of muscle wasting diseases.

### Laminin α2 Chain-Deficient Mice Overexpressing Transgenes or Lacking Other Genes

The different LAMA2-CMD mouse models have been vital tools for the identification of disease driving mechanisms and for developing therapeutic approaches. *Dy/dy, dy^2J^/dy^2J^*, *dy*^W^/*dy*^W^, and *dy*^3K^/*dy*^3K^ mice have been genetically manipulated to overexpress or knockout specific genes that were hypothesized to impact disease pathogenesis or serve as suitable therapy candidates (Tables 4, 5). Moreover, successful gene editing by CRISPR-Cas9 has been performed to correct the splicing defect in *dy*^2J^/*dy*^2J^ animals (Kemaladewi et al., 2017; Table 4). Correcting the primary underlying abnormality, which is loss of laminin α2 chain, and amending the subsequent disruption of the linkage between the basement membrane and the cytoskeleton, is probably the most attractive therapeutic goal for LAMA2-CMD. Consequently, laminin α2 and α1 transgenes, respectively, have been overexpressed in *dy*^W^/*dy*^W^,*dy*^3K^/*dy*^3K^, and *dy*^2J^/*dy*^2J^ mice, conferring excellent amelioration of the dystrophic phenotype (Kuang et al., 1998b; Gawlik and Durbeej, 2010; Gawlik et al., 2004, 2018; Table 4). More recently, overexpression of laminin α1 chain in *dy*^2J^/*dy*^2J^ mice was achieved with an adeno-associated virus carrying a catalytically inactive Cas9 with VP64 transactivators and single guide RNAs that target the *Lama1* promoter (Kemaladewi et al., 2019; Table 4).

The primary defect of the disease was also targeted through clever molecular strategies that aimed at restoring the linkage between the extracellular matrix and cytoskeleton without the necessity of introducing the whole laminin α2 or α1 chain. Transgenic or AVV-mediated overexpression of mini-agrin alone (Moll et al., 2001; Qiao et al., 2005) or in particular mini-agrin transgene in combination with αLNNd transgene (a laminin/nidogen chimeric protein) (Reinhard et al., 2017) resulted in superb skeletal muscle restoration in *dy*^W^/*dy*^W^ mice (Table 4). Mini-agrin together with the anti-apoptotic Bcl-2 transgene also profoundly reduced muscular dystrophy in *dy*^W^/*dy*^W^ mice (Meinen et al., 2011; Table 4). Moreover, mini-agrin overexpression has been evaluated in *dy*^3K^/*dy*^3K^ mice (very good muscle restoration) (Bentzinger et al., 2005) but not the more attractive combination of mini-agrin and αLNNd. Additionally, αLNNd overexpression alone was shown to correct muscular dystrophy in *dy*^2J^/*dy*^2J^ animals (McKee et al., 2017; Table 4).

Xu et al. (2007) tested another way to modulate the extracellular matrix in *dy*^W^*dy*^W^ mice. Cytotoxic T cell GalNAc transferase is an acetylgalactosaminyl-transferase that creates a CT-carbohydrate on selected glycoproteins and glycolipids. When overexpressed extrasynaptically in *dy*^W^*dy*^W^ muscle, muscular dystrophy was reduced (Table 4).

Genetic manipulations have also been used to evaluate the roles of the laminin α2 chain receptor integrin α7 and the members of the dystrophin-glycoprotein complex (DGC) in the LAMA2-CMD disease pathology in the different mouse models. Overexpression of integrin α7 (that is absent from the sarcolemma in LAMA2-CMD muscle) reduced muscular dystrophy in *dy*^W^/*dy*^W^ mice (Doe et al., 2011; Table 4). Deletion of integrin α7, on the other hand, did not aggravate the disease symptoms in *dy*^3K^/*dy*^3K^ mice, indicating that laminin α2 chain and integrin α7 have complementary functions in skeletal muscle (Gawlik and Durbeej, 2015; Table 5). In contrast, deficiency of dystrophin and β-sarcoglycan, respectively, severely worsened the phenotype of *dy*^3K^/*dy*^3K^ mice (Gawlik et al., 2014; Table 5). These results suggested non redundant roles of laminin α2 and the DGC and a key impact of laminin-DGC axis on muscle homeostasis. At the same time, studies with *dy*^3K^/*dy*^3K^ mice overexpressing laminin α1 chain with preserved integrin α7 binding domains but lacking dystroglycan binding sites emphasized the significance of both linkages in rescuing the dystrophic phenotype in muscle (Gawlik et al., 2010; Table 4).

As the dystrophic LAMA2-CMD pathology is very complex, a great deal of effort has been aimed at elucidating the secondary pathogenic mechanisms. For example, augmented apoptosis, proteasomal activity and autophagy as well as impaired mitochondrial function, excessive inflammation and pathological fibrosis are all major disease drivers in LAMA2-CMD (see also section “Cellular and Molecular Events in Murine Laminin α2 Chain-Deficient Muscle”). Accordingly, genetic approaches that target some of these secondary pathologies have been assessed in the different mouse models. Both transgenic overexpression of the anti-apoptotic protein Bcl2 and deletion of the pro-apoptotic protein Bax reduced muscular dystrophy in *dy*^W^/*dy*^W^ mice (Girgenrath et al., 2004; Tables 4, 5) and removal of the mitochondrial calcium regulator cyclophilin D, which regulates mitochondrial permeability transition pore, attenuated muscular dystrophy in *dy*^W^/*dy*^W^ mice (Millay et al., 2008; Table 5).

As LAMA2-CMD skeletal muscle is characterized by early acute inflammation and subsequent fibrosis, a few studies have investigated the roles of certain pro-inflammatory and pro-fibrotic molecules in disease pathogenesis. Deletion of osteopontin and galectin-3 (both involved in inflammatory and fibrotic processes), respectively, did not reduce muscle pathology in *dy*^3K^/*dy*^3K^ mouse. In fact, removal of osteopontin slightly worsened the phenotype indicating that osteopontin might be a beneficial immunomodulator in LAMA2-CMD (Gawlik et al., 2017). Similarly, absence of pro-fibrotic miR-21 in *dy*^3K^/*dy*^3K^ and *dy*^2J^*dy*^2J^ mice did not improve muscular dystrophy (Moreira Soares Oliveira et al., 2017). Complement 3-deficiency, on the other hand, prolonged survival in *dy/dy* mice (Connolly et al., 2002; Table 5). Although genetic manipulations of osteopontin, galectin-3 and miR-21 in LAMA2-CMD mice did not reveal any major impact on development on fibrosis, pharmacological treatment with compounds that target inflammation and fibrosis has successfully been employed in *dy*^W^/*dy*^W^ and *dy*^2J^/*dy*^2J^ mice (these compounds and other pharmacological strategies will briefly be described below in section “Pharmacological Approaches in the Mouse Models for LAMA2-CMD”).

Moreover, several attempts to boost regeneration in LAMA2-CMD mouse models have been performed. For example, transgenically overexpressed IGF-1 very well improved the outcome of *dy*^W^/*dy*^W^ mice (Kumar et al., 2011; Table 4). In contrast, overexpression of ADAM12 or removal of myostatin, respectively, did no reduce muscular dystrophy in *dy*^W^/*dy*^W^ mice (Guo et al., 2005; Li et al., 2005; Tables 4, 5).

Finally, Kemaladewi et al. (2018) recently identified imbalanced polyamine metabolism in *dy*^2J^/*dy*^2J^ tibialis anterior muscle and developed a strategy to increase the polyamine level by lentiviral-mediated overexpression of adenosylmethionine decarboxylase (Amd1) and spermine oxidase (Smox) (Table 4).

### Pharmacological Approaches in the Mouse Models for LAMA2-CMD

The genetic interventions described above are undoubtedly important for the development of therapeutic approaches for LAMA2-CMD. Yet, the translation of several of these lines of attack into clinical practice remains challenging. For this reason, a number of pharmacological approaches have been investigated in the different mouse models and could ultimately permit clinical treatment possibilities. The approaches include targeting both the primary gene deficiency as well as the secondary disease drivers. Burkin and co-workers have elegantly demonstrated that laminin-111 protein therapy reduces muscular dystrophy and improves muscle repair in **dy*^W^/*dy*^W^*mice (Rooney et al., 2012; Van Ry et al., 2014). Apoptosis inhibition has also been evaluated with pharmacological compounds. Doxycycline and omigapil, respectively, decreased muscle pathology in **dy*^W^/*dy*^W^* mice and omigapil also had beneficial effects in **dy*^2J^/*dy*^2J^* mice (Girgenrath et al., 2004; Erb et al., 2009; Yu et al., 2013). Similarly, proteasome inhibition with MG-132 and bortezomib, respectively, and autophagy inhibition with 3-methyladenine amended some of the pathological features in **dy*^3K^/*dy*^3K^* mice (Carmignac et al., 2011a, b; Korner et al., 2014). Additionally, compounds that modulate inflammation (glatiramer acetate, prednisolone) (Connolly et al., 2002; Dadush et al., 2010; Rabie et al., 2019) and anti-fibrotic compounds (halofuginone, losartan, and losartan derivative) have been shown to diminish muscle pathology in **dy/dy**, **dy*^2J^/*dy*^2J^*, and**dy*^W^/*dy*^W^* mice (Nevo et al., 2010; Elbaz et al., 2012; Meinen et al., 2012; Vohra et al., 2015). Lastly, compounds that modulate muscle growth (clenbuterol), calcium levels (caldecrin), metabolism (metformin), oxidative stress as well as exon skipping with phosphoroamidate morpholino oligomers have all shown a positive influence on the muscle phenotype in the different LAMA2-CMD mouse models (Hayes and Williams, 1998; Tomomura et al., 2011; Aoki et al., 2013; Fontes-Oliveira et al., 2018; Harandi et al., 2020; Table 6).

Considering that many different cellular functions are dysregulated in LAMA2-CMD, there have been a few reports describing strategies that simultaneously target diverse processes. Indeed, it was demonstrated that amelioration of pathology was greater with a combination of mini-agrin and Bcl-2 transgenes than single mode therapies. Similarly, a combination of mini-agrin and omigapil resulted in excellent muscle condition (Meinen et al., 2011; Tables 4, 6). Girgenrath and co-workers have also evaluated combinatorial treatment and demonstrated that a combination of IGF-1 transgene and removal of Bax profoundly ameliorated disease pathology in *dy*^W^/*dy*^W^ mice and so did removal of Bax in combination with systemic recombinant human IGF-1 (IPLEX) (Yamauchi et al., 2013; Tables 4–6). Moreover, a combinatorial treatment utilizing transgenic IGF-1 in conjunction with losartan led to remarkable reduction of muscular dystrophy in *dy*^W^/*dy*^W^ mice and also growth hormone enhanced losartan treatment in *dy*^W^/*dy*^W^ mice (Accorsi et al., 2016; Table 6).

## Discussion

Mouse models for LAMA2-CMD have facilitated our understanding of the disease for over two decades. During that time the research community has tried to address important disease-related questions: from the basic phenotype description, through strategies to prevent the disorder by the most obvious transgenic means (introducing the laminin α2 and α1 transgenes) or the more sophisticated molecular manipulations (combination of engineered transgenes, genome editing with CRISPR/Cas9), through pharmacological approaches to target the disease symptoms.

We are now facing a number of questions that need to be answered in order to advance the research on LAMA2-CMD. What is next? How can we use the available animal models in the best possible way? What should we focus on? Would it be justified and feasible to mimic the disease in a bigger animal? Should we increase our interest in Drosophila, nematodes, zebrafish, frogs, and newts? Indeed, Peter Currie’s work in zebrafish has substantially contributed to understanding of pathogenic mechanisms of the disease (Gupta et al., 2012; Sztal et al., 2012; Hall et al., 2019; Wood et al., 2019).

Is there a need for creating additional mouse models for LAMA2-CMD? For example, mice with a tissue-specific deletion of the *Lama2* gene would surely become an asset in LAMA2-CMD research. Perhaps one important task would be to identify mutations in existing mouse models (*dy/dy* and *dy*^6J^/*dy*^6J^). Additionally, we should further explore the new methods and technologies that in a robust way contribute to reliable evaluation of preclinical treatment outcomes. For example, magnetic resonance imaging, electrical impendence myography and identification of biomarkers could complement the classical histopathology evaluations and functional tests (Accorsi et al., 2015; Vohra et al., 2015; Moreira Soares Oliveira et al., 2018; Nichols et al., 2018).

The reproducibility of data and well-grounded comparison of outcomes are a key to good science. One of the crucial tasks that we need to dedicate more energy to is a seemingly trivial, low-status assignment to create the standard operating procedures (SOPs) for all mouse models that all researches agree upon. Another task is to… stick to them: it is still a common practice that researchers follow their own protocols despite SOPs availability. SOPs have been generated for some of the mouse models for various muscular dystrophies/muscle diseases^4^. Some of these protocols can be easily adapted to LAMA2-CMD mouse models (Gawlik et al., 2019). It is also encouraging that several SOPs have been described for *dy*^W^/*dy*^W^ mouse model^5^. Hence, we head toward the right direction, but the researchers in the LAMA2-CMD field need to step up and focus further on this issue. For example, SOPs for *dy*^3K^/*dy*^3K^ and *dy*^2J^/*dy*^2J^ mouse models should be created.

Variability in growth rates and overall survival of *dy*^W^/*dy*^W^ animals from different laboratories have been noted (Willmann et al., 2017) and such discrepancies are likely to occur for the other mouse models as well. Therefore, it may be highly relevant to replicate the different therapeutic strategies in different mouse models and in different laboratories (even though such studies will be difficult to publish). It is also essential to thoroughly analyze the onset of pathological features in the variety of muscles in LAMA2-CMD mouse models in order to choose optimal time-points for preclinical interventions (despite the fact that most patients today may not be treated before the disease onset in the corresponding clinical set up) and select the most relevant muscles for treatment evaluation.

Thus far we face an incomplete picture of pathology in different muscle types. Considering that only in the hind limb and pelvis of a mouse there are 39 muscles (Charles et al., 2016), very few muscles have been analyzed in LAMA2-mouse models. What is more, only selected dystrophic features (for example, only fibrosis) and a limited range of time-points have been described in most publications (see references in Tables 4–6). Therefore, assessments of muscle phenotypes lack a quantitative aspect and are often based on general morphology and a researcher’s perception. Again, potential studies that aim at filling those gaps do not align with current publishing and funding policies. As a result, they are rarely prioritized. Coordinated effort between different laboratories should be undertaken to focus on quantitative methods, time points and muscles of choice. In general, natural history studies, standardization of outcome measures, reporting of negative findings and importance of data validation should be further emphasized. Notably, the researchers are not on their own in these efforts. Patients’ organizations (for example, Cure CMD^6^) understand the significance of such studies and greatly support basic research.

Exploring treatment strategies in a preclinical setup is a challenging and attractive goal for scientists that work with animal models. However, it has become clear that deeper understanding of pathogenic mechanisms underlying the disease development needs to be focused on. In particular, signaling pathways and involvement of different cell types in LAMA2-CMD pathology could be crucial for therapy design. Apart from well-known abnormalities in the central and peripheral nervous system (that are manifested both in LAMA2-CMD patients and mouse models) (Chun et al., 2003; Quijano-Roy et al., 2012; Bönnemann and Voermans, 2012; Bonnemann et al., 2014; Menezes et al., 2014), more subtle extramuscular defects are evident in mice (e.g., hearing loss, impaired spermatogenesis, and aberrant development of thymocytes and odontoblasts) (Magner et al., 2000; Pillers et al., 2002; Yuasa et al., 2004; Hager et al., 2005). These aspects of LAMA2-CMD together with the central nervous system manifestations have been poorly studied. Similarly, other tissues that normally express laminin α2 chain (heart, smooth muscle) have not been fully evaluated.

Altogether, animal models are an attractive platform to investigate the variety of the disease symptoms and treatment strategies. Nevertheless, it is important to bear in mind that relatively few preclinical findings will be successfully translated to humans as mice do not always truthfully model human disease pathology. This diminishes the predictive value of animal-based discoveries for future clinical studies (Gawlik, 2018). But if only one sole finding is positively verified and could be implemented in humans, it would be a huge achievement. It all boils down to cumulated effort to properly validate preclinical results and carefully choose targets for clinical trials. That is especially important for LAMA2-CMD where a limited pool of patients for clinical test is available. The take-home message is that we have to keep going: even if the alternatives for animal research become more and more useful (induced pluripotent stem cells, human cell-based assays), they suffer from various limitations: they cannot mimic multiple interactions between various tissues, organs and immune system. In addition, pharmacokinetic/pharmacodynamic modeling is not feasible using these methods. Consequently, they are not robust enough to provide reliable verification platform for such a complex disease. Hence, the mouse models still offer the best chance for important discoveries and preclinical studies for muscular dystrophies like LAMA2-CMD will certainly remain fully dependent on animals.

## Conclusion

In summary, we believe that research opportunities on the mouse models for LAMA2-CMD will continue to inspire us, scientists, and spur our joint effort to design effective treatment for the disease.

## Author Contributions

KG and MD wrote the manuscript and secured funding.

## Conflict of Interest

The authors declare that the research was conducted in the absence of any commercial or financial relationships that could be construed as a potential conflict of interest.

## Footnotes

1 https://www.jax.org/strain/013786

2 https://www.jax.org/strain/003589

3 https://www.jax.org/strain/009692

4 https://treat-nmd.org/research-overview/preclinical-research/

5 https://treat-nmd.org/research-overview/preclinical-research/sops-for-cmd-animal-models/

6 www.curecmd.org

## Supplementary Material

VIDEO S1 A 3-week-old *dy*^3K^/*dy*^3K^ mouse and a wild-type littermate. The *dy*^3K^/*dy*^3K^ mouse is not active when placed in a new cage.
